# Can hybrid educational activities of team and problem based learning program be effective for Japanese medical students?

**DOI:** 10.5116/ijme.591b.2bc8

**Published:** 2017-05-16

**Authors:** Kentaro Iwata, Asako Doi

**Affiliations:** 1 Division of Infectious Disease, Kobe University Hospital, Japan; 2 Division of Infectious Diseases, Kobe City Medical Center General Hospital, Japan

**Keywords:** Hybrid Educational Activities of TBL and PBL Program (HEATAPP), team based learning (TBL), PBL, Japan

## Introduction

Active learning such as problem based learning (PBL) and team based learning (TBL) has become increasingly popular in medical education among Asian countries including Japan.^1^^,^^2^ We developed a novel learning method named Hybrid Educational Activities of TBL and PBL Program (HEATAPP), which incorporated characteristics of both TBL and PBL, since we considered that employing TBL has the advantage of requiring less faculty members, of which we have a shortage. Additionally, PBL can strengthen clinical reasoning skills, which we consider is one weakness our students have. However, its effectiveness has never been evaluated. We held a focus group discussion and interviews for 6 medical students who participated in HEATAPP, and we found its strengths and weaknesses, particularly for Japanese students who might have unique characteristics different from other nations. Here we review what we found and try to envision what should be done.

### Overview of the course

HEATAPP has been provided to all of 4th year medical students since 2012. By the time HEATAPP was held, the students had been through all basic medicine curricula and they had completed a medical English course.

The participants were divided into teams of about 6 people. HEATAPP does not require readiness assurance activities as in conventional TBL, to focus more on clinical reasoning. We rather discussed the case without pre-notifying what the case is about, as in the PBL of Hawaii University, from which we adopted a lot.^3^

After listening to a few sentences of the presenting illness of a case (patient’s age, sex and chief complaint), a group discussion on clinical reasoning, hypothesis generation and verification of the developed hypotheses was encouraged. The tutor (KI) provided further clinical information little by little (working forward), not revealing all information at once, to avoid thinking “backward”.^4^ The participants continued to discuss the case by alternating the mini-lectures from the tutor with small group discussions until reaching their final assessment of the case. Unlike conventional PBL, the tutor actively joined discussion and taught knowledge, skills, or principles of management of each case.

After these activities, the participants were asked to develop a Your Specific Question (YSQ) per team. YSQ was to investigate a topic regarding the case, mostly on diagnosis, treatment, epidemiology, or pathogenesis, which students chose to study.

After deciding YSQ, the session was adjourned and each group was asked to solve their YSQ. Group work on each YSQ was presented on the following morning, and the next new case was discussed afterward.

Students dealt with one case a day, dealing with a total of 5 cases. On the 4th and 5th day, cases were presented in English to encourage the use of English to the students.

### What we found through group discussion

After reviewing the group discussion, we found that many felt HEATAPP was effective in active learning, group discussion, and developing hypotheses and questions.

However, some students also expressed difficulty in active, participatory learning. For example, some preferred classical didactic lectures and memorization of things. In addition, some complained of not having much teaching about treatment, which we expected the students to learn through YSQ. Many also felt that asking questions was difficult. They often ended up with easy questions to answer, which might not be clinically important. They also found working in English very difficult.

We also found there were some less motivated students in teams. They merely relied on so-called “super-achievers”, copying and reading out the manuscripts these super-achievers prepared, and essentially did not participate in the activities at all.

They also found the difference between Japanese system and systems in other countries. The implication is that Japanese medical students are burnt due to the entrance exam (Juken) and need time to relax after entering medical schools.

### What went wrong with HEATAPP? What should be done?

We found several problems in HEATAPP after the group discussion. Even though active learning has gained popularity, it can be difficult to execute, particularly among students who are so used to learning things passively. This seems particularly true in Asian countries with the influence of Confucianism, where people are used to learning things through didactic lectures, spoon feeding knowledge and memorizing without any critique.^1^^,^^5^

PBL-incompatible Asian cultural attitudes include fear of confrontation with authority, distaste for open criticism of authority, Confucian socialization requiring a person not to be outspoken, lack of passion for studies, lack of ability/motivation to ask questions, and low participation in class discussion.^1^ Some argue that the passive attitudes of Asian students are not inherent to them, and it is more due to situation-specific factors such as teaching methodologies.^5^ Whether this view holds true or not, the learning habits of passive learning are indeed preventing them from learning actively. Culturally inherited or not, we need to alter the way we are.

Many medical students in Japan misunderstand PBL as problem solving, instead of learning.^6^ Students try to find the diagnosis as “the answer”, the manoeuvre many Japanese medical students are very good at (guessing diagnosis right game). PBL in Japan is often done by “thinking backward”,^4^ providing all clinical information at once, and astute students search the internet using keywords to find the “answer” quickly. This, however, does not lead to better understanding of real patients in clinical practice.

To make things worse, some students are already burnt out after the rigor of entrance examinations. After years of training at school and knowledge-cramming schools, medical students seek happiness in extra-class activities and they float through their school years.^7^ Since medicine is an ever progressing field and its progress is getting faster and faster, life long active learning by physicians is a must. Therefore, the tendency of burning out and not studying after entering medical schools needs significant reform. Assigning homework or examinations might improve the attitude of those less motivated, but these will not increase their motivation (if not the opposite).

To overcome these problems underlying Japan’s medical schools, we probably need tactics to lead them to learn actively and lifelong. This means that just imitating western countries is not the answer (and imitation is ironically a kind of passive learning). For short-term improvement, one needs some gradual transition from passive to active learning. Teaching why active learning is important is another way to reconcile these two types of learning. For the long-term, Japanese students need to learn how to learn actively beginning in their childhood. This means we need fundamental reform in the educational system.

Lack of English skills, despite rigorous “Juken” study is another issue to overcome. The inability to search and read literature (most of which are written in English) is a drawback in active learning and information management, and physicians in the 21st century would not survive without these skills. In HEATAPP, we discuss cases in English for 2 days, but it is not enough in making students familiar with English. Long term practical use of English and making them read and write in English easily is an extremely important task.

## Conclusions

We invented a novel educational activity called HEATAPP for active participatory learning for Japanese medical students. However, because of long term dependence on passive learning, implementing active learning might cause resistance among students. Some students are burnt out by the time they entered medical schools and group studies might be manipulated to allow them to be indolent. Lack of English skill is another impediment to search information actively.

We probably have to introduce active learning more carefully to make Japanese medical students more accustomed to this type of learning they are not familiar with. Introduction of active learning early in childhood would be a fundamental, long-term solution to this problem.

### Acknowledgements

The authors truly appreciate Dr Daniel J Mosby for correcting English.

### Conflict of Interest

The authors declare that they have no conflict of interest.
